# Recommendations for Implementing Gamification for Mental Health and Wellbeing

**DOI:** 10.3389/fpsyg.2020.586379

**Published:** 2020-12-07

**Authors:** Vanessa Wan Sze Cheng

**Affiliations:** Brain and Mind Centre, The University of Sydney, Sydney, NSW, Australia

**Keywords:** engagement, wellbeing, mental health, mHealth, eHealth, gamification, applied games, health information technologies

## Abstract

Gamification is increasingly being proposed as a strategy to increase engagement for mental health and wellbeing technologies. However, its implementation has been criticized as atheoretical, particularly in relation to behavior change theory and game studies theories. Definitions of the term “gamification” vary, sometimes widely, between and within academic fields and the effectiveness of gamification is yet to be empirically established. Despite this, enthusiasm for developing gamified mental health technologies, such as interventions, continues to grow. There is a need to examine how best to implement gamification in mental health and wellbeing technologies in a way that takes quick production cycles into account while still emphasizing empirical investigation and building a rigorous evidence base. With reference to game studies and the medical (eHealth/mHealth) literature, this article interrogates gamification for mental health and wellbeing by examining core properties of the game form. It then explores how gamification can best be conceptualized and implemented for mental health and wellbeing goals from conceptualization through to iterative co-development and evaluation that accommodates software development schedules. Finally, it summarizes its conceptual analysis into recommendations for researchers and designers looking to do so. These recommendations are: (1) assess suitability, (2) implement to support, (3) assess acceptability, (4) evaluate impact, and (5) document comprehensively. These recommendations aim to encourage clear language, unified terminology, the application and evaluation of theory, comprehensive and constant documentation, and transparent evaluation of outcomes.

## Introduction

### Digital Health Technologies and the Engagement Problem

Digital health technologies, such as mobile phone apps and Web-based interventions, are increasingly considered a cost- and resource-efficient method of delivering health interventions to the general population. They can be accessed from any location in the world with an Internet connection, and as such have the potential to overcome geographical, awareness, attitudinal, and potentially even financial barriers to access (Price et al., 2014). The flexibility of these technologies also means they can be deployed at any part of the treatment timeline (pre-, during, and post-treatment) and can serve a variety of roles such as education (including psychoeducation and skills training), symptom tracking, distraction from pain or unpleasant emotions, and communicating remotely with a therapist (Price et al., 2014). In tandem with face-to-face consultations and treatments, digital health technologies have the potential to fundamentally restructure the healthcare system.

However, while clinical evaluations of eHealth technologies have found beneficial effects on mental health and wellbeing (Spijkerman et al., 2016), potentially resulting in better outcomes than their face-to-face counterparts (Lappalainen et al., 2014), they have also observed considerable attrition rates. Notably, a review of Web-based interventions aimed at common mental disorders found highly variable rates of adherence to study protocol ranging from 3.37 to 100% (Brown et al., 2016b). Attrition rates increase, and adherence rates decrease, further once the technology is rolled out for public usage (Fleming et al., 2016). For example, a study comparing module completion in an online cognitive behavioral therapy intervention found that 66% of trial participants had completed two or more modules of the program, compared to only 15.6% of community participants (Christensen et al., 2004).

A proactive strategy of encouraging users to make contact, and then continually engage, with interventions has been linked to decreased attrition (Kelders et al., 2012). One strategy that has been proposed and employed in eHealth and mHealth to encourage such engagement and re-engagement is gamification (Deterding et al., 2011; Huotari and Hamari, 2012; Cugelman, 2013). This article interrogates the concept of gamification for mental health and wellbeing and provides research and design recommendations synthesized from the literature as well as a doctoral project that involved the co-design, development, and evaluation of a gamified mental health and wellbeing app.

### Defining Gamification for Mental Health and Wellbeing

While the term “gamification” has been used to describe multiple game-related concepts in the past, recent academic consensus has settled on using it to describe the process that Deterding et al. (2011) define as “the use of game design elements in non-game contexts.” Real-world examples of gamification include the Nike+ system, which aims to promote regular running through socially competitive mechanics, and Code Academy, which rewards users who complete its educational courses with points and badges (Sicart, 2014).

Despite the growing consensus on what “gamification” describes, inconsistencies still exist in the literature (as observed by Seaborn and Fels, 2015). This may be partially due to gamification’s explosive popularity, and the wide variety of disciplines from which its users and researchers come, all of whom approach it with their own perspective and framing. The term “gamification” is often used to describe other closely related forms of applied games, such as serious games (Seaborn and Fels, 2015). However, by examining the Deterding et al. (2011) definition, we can more precisely delineate the differences between gamification and other forms of applied games. Specifically, Deterding et al. (2011) position “play” and “games” in opposition to each other. This is consistent with sociologist Roger Caillois’ conception of play as a spectrum between “*paidia*” (free, unstructured play) and “*ludus*” (rules-based, goal-directed play—that is, games). On a continuum of game vs. play and whole vs. parts, Deterding et al. (2011) situate gamification (also referred to as “gameful design”) as partly game. This is in contrast to games, including serious games, which are wholly game. Gamification is also positioned as conceptually opposite to toys (wholly play) and related, but distinct, to playful design (partially play).

Deterding et al.’s (2011) definition is frequently cited in calls for applying gamification for health and wellbeing (e.g., Cugelman, 2013; King et al., 2013). However, alternate definitions for gamification exist. Huotari and Hamari (2012) propose one that is particularly suited for application to health and wellbeing, including mental health: “a process of enhancing a service with affordances for gameful experiences in order to support [a] user’s overall value creation” (p. 20). By placing the emphasis back on the *service* (Huotari and Hamari use this term in a general goods and services context), this definition complements the goals of health services research. Furthermore, unlike Deterding et al.’s definition, which implies that gamification is a property (i.e., a technology is gamified), Huotari and Hamari’s definition implies that gamification cannot occur without a gameful experience: that is, without the perception of such from the user. This will be discussed in more detail later in this article. Nevertheless, both definitions provide useful ways with which to conceptualize gamification. While Deterding et al.’s definition emphasizes the *elements* of game design and is therefore useful as a (taxonomic) lens through which to approach *researching* gamification, Huotari and Hamari’s definition is useful as a lens through which to *implement* it.

### Briefly Reviewing the Effectiveness of Gamification for Mental Health and Wellbeing

Gamification is experiencing increasing application in digital health, often in the form of badges, leaderboards, points, and challenges (Miller et al., 2014). In the field of physical health, it is commonly applied to physical fitness and diet (Lister et al., 2014) as well as chronic illnesses (Lazem et al., 2015; AlMarshedi et al., 2017; Sardi et al., 2017). Notably, Lister et al.’s (2014) review on physical fitness and diet mobile phone apps found that gamification was present in just over half of the sampled apps, and that just under a quarter of their sample contained more than three gamification elements (as defined by the authors).

However, compared to physical health, there seems to be less uptake of gamification for mental health and wellbeing (Johnson et al., 2016). While it has been applied to mood and resilience (Roepke et al., 2015; Litvin et al., 2020), anxiety disorders (Dennis and O’Toole, 2014; Miloff et al., 2015; Lindner et al., 2020), tobacco and substance misuse (Earle et al., 2018; Struik et al., 2018; Zhang et al., 2019), sleep (Werner-Seidler et al., 2017), wellbeing (Vella et al., 2018), and serious mental illness (Varnfield et al., 2019), gamified mental health interventions tend to include less gamification features, with a systematic review finding the majority of included interventions used only one (Brown et al., 2016b). Another review on stress management apps in the Google Play Store also found low levels of gamification (Hoffmann et al., 2017). Specifically, only 32% of the sampled apps employed gamification, and the apps that did use gamification tended to contain only one gamification element (as defined by the authors). This field enjoys a relatively quick pace of change, with a more recent systematic review by Cheng et al. (2019) finding that the median number of gamification elements used in the included gamified mental health apps was 5. However, it must be noted that these three reviews defined gamification elements differently.

This article focuses primarily on gamification for mental health and wellbeing. However, the relative lag in uptake of gamification for mental health means that mental health gamification literature is less comprehensive. For this reason, I will discuss gamification for general health and wellbeing for the remainder of this section while noting mental health-specific research where available.

There is increasing interest in using gamification for health purposes, particularly to target low engagement with health technologies and improve adherence to health behaviors. However, gamification is often applied without fully considering engagement, motivation, or behavior change theories (Seaborn and Fels, 2015). For example, a review by Lister et al. (2014) of gamified health and fitness apps, through the lens of the health behavior change wheel (Michie et al., 2011), found that the gamification in their sample overwhelmingly focused on motivational drivers of health behavior change, when in fact a focus on capability and opportunity drivers is also required. Similarly, a systematic review of gamification in health and wellbeing by Johnson et al. (2016) found that the majority of included studies described a behaviorist implementation of gamification, with little consideration of intrinsic motivation. Additionally, a systematic review by Cheng et al. (2019) used relatively broad criteria to capture researcher justification for including gamification in their apps or technologies for mental health, and found that 41% (39/70) of the papers in their sample did not provide any reason for doing so. More recently, Schmidt-Kraepelin et al. (2020) conducted a systematic review of studies investigating health behavior change theories and gamification and found that of the 25 papers reviewed, seven only briefly mentioned a health behavior change theoretical framework, and only five had fully integrated a health behavior change theory into the gamified technology.

As gamification is still an emerging area of inquiry, the majority of research is exploratory and solution-focused instead of evaluative. There is a relative lack of research into how effective gamification is (Sardi et al., 2017), with definitions of effectiveness naturalistically broad due to the breadth of domains gamification is researched in. A descriptive review of empirical studies on the effects of gamification (mostly in the fields of computer science, education, and management science) suggests that the implementation of gamification has positive general effects; however, the authors also suggest that this could potentially be due to a novelty effect, and that the removal of gamification could induce loss aversion (not wanting to lose already earned badges and points) and alienate currently engaged users as a result (Hamari et al., 2014). The authors also note possible confounding effects of the context of gamification and individual differences between users, and that the effects of gamification can be more complex than is assumed.

Within the health literature, a review of games (the authors included gamification in their review) applied to diabetes could not draw a conclusive relationship between the usage of gaming concepts and clinical health outcomes (Lazem et al., 2015). A review of gamification for health and wellbeing, however, found preliminary evidence broadly suggesting a positive impact of gamification, particularly on mental wellness (Johnson et al., 2016). Another review on Web-based mental health interventions containing gamification found no significant overall difference in rates of adherence to interventions based on number of gamification features incorporated; however, the authors were limited by a lack of detail in reporting (of both adherence and gamification) in the papers reviewed (Brown et al., 2016b). In comparison, a review on physical fitness and diet mobile phone apps found that while the presence of game elements (as defined by the authors) was associated with app popularity (as quantified by the number of app reviews), the presence of gamification (again, as defined by the authors) was not (Lister et al., 2014). Lister et al. argue that this could potentially be due to inappropriate and/or incomprehensive application of gamification strategies, such as a poor balance between the effort needed to obtain a reward and the value of the reward itself. Finally, Floryan et al. (2020) found degree of implementation of gamification principles to correlate with app quality and app store rating. There is also little to no evidence on whether the effects of gamification persist in the long term (Cugelman, 2013; Sardi et al., 2017). In response, there have been calls for stronger evaluations of the effectiveness of gamification (Seaborn and Fels, 2015; Hoffmann et al., 2017).

Current evidence for the general effectiveness of gamification for health and wellbeing is, therefore, inconclusive. However, studies empirically testing individual elements of gamification have produced results more strongly suggestive of beneficial effects. For example, in a relatively large-sample (*n* = 1,162), between-subjects study, Comello et al. (2016) found that using game-inspired feedback formats (progress bars and scorecards) across various health domains (e.g., tobacco use, physical activity) led to higher comprehension and engagement outcomes in certain cases and non-inferior outcomes in others, supporting the adoption of this particular format of behavioral feedback. Similarly, there is evidence that the presence of badges (Hamari, 2017) and social comparison (measured through social media-esque “likes”; Hamari and Koivisto, 2015) are individually associated with greater engagement with a gamified service. By operationalizing gamification as the presence, or absence, of certain elements, these studies are able to directly attribute group differences between conditions to these elements. However, as a result they also do not explicitly account for the user perception of a gameful experience. In other words, it is not clear whether these studies’ participants would perceive the difference between the experimental and control conditions as being more or less gamified.

Other research suggests that the effectiveness of individual gamification elements is also affected by the psychological context of the gamified technology. In their study comparing different versions of a pedometer app containing different functionalities, Zuckerman and Gal-Oz (2014) report that while a “quantified” version providing behavioral feedback outperformed baseline (the app in an inactive state with no functionality or interactions), “gamified” versions of the quantified app that added either virtual rewards (points) or social comparison (a leaderboard) did not outperform the quantified version. Similarly, an interview study by Helmefalk et al. (2020) found that participants appeared to find the data tracking capabilities of their physical activity trackers more fulfilling than the gamification aspects (badges, fireworks, and social media sharing), as the former supported their basic needs of autonomy, competence, and relatedness (from self-determination theory; Deci and Ryan, 2000). Interviews with participants in both studies found that most participants did not see the gamification elements as meaningful, with Helmefalk et al. (2020) suggesting that this could be because the added gamification elements did not directly address the basic needs of their participants. This once again suggests that gamification should be applied in a more theory-driven manner, and that more game mechanics beyond the commonly seen points, badges, and leaderboards be explored to deliver the intrinsically motivating gamelike experience that the term and its definitions promise. However, this does not mean that extrinsic motivators should be eschewed altogether, but rather that they should be implemented in ways that do not thwart feelings of competence or autonomy (Loughrey and Broin, 2018).

So far, it is clear that different studies from different academic fields (and sometimes even from the same field) conceptualize gamification differently. Establishing not just a consistent definition, but also operationalization, of gamification would empower its application and evaluation by multiple researchers across academic fields.

## Interrogating Gamification for Mental Health and Wellbeing

### Understanding Gameful Experiences Through Understanding Games

Huotari and Hamari’s (2012) definition of the term “gamification” implies that for gamification to occur, a gameful experience must be had. What, then, is a gameful experience?

Landers et al. (2019) define a “gameful experience” as an “interactive state occurring when a person perceives non-trivial achievable goals created externally, is motivated to pursue them under an arbitrary set of behavioral rules, and evaluates that motivation as voluntary.” In other words, they view it as a formative psychological construct made up of goal perception, rule endorsement, and voluntary motivation. Landers et al. also emphasize the difference between gameful design, gameful systems, and gameful experiences, and propose in detail a multilevel (system and individual) model of gamefulness that connects these three constructs with behavior change moderated by individual differences. By isolating out the gameful experience as a construct separate to gameful design and systems, Landers et al. emphasize that it needs to be measured as a potential mediator of any impacts of gameful design (e.g., on adherence or mental health). As of the time of writing, however, a measure of gameful experience that aligns with their model of gamefulness has yet to be developed and validated.

Other work on developing instruments for measuring gameful experience focuses more on the characteristics of the actual experience (as opposed to the psychological characteristics that lead to it) and suggests that it is a multidimensional construct. In their work, Eppmann et al. (2018) identify six factors (enjoyment, absorption, creative thinking, activation, absence of negative effect, and dominance), while Högberg et al. (2019) identify seven (accomplishment, challenge, competition, guided[ness], immersion, playfulness, and social experience). However, while this research has established the importance of these constructs to the gameful experience, the argument can also be made that artifacts without these traits can still be considered a game (Stenros, 2016). Essentially, there is more at play. This article will, therefore, proceed to briefly review relevant literature from the field of game studies.

In contemporary culture, the word “game” most saliently conjures up impressions of digital games (also known as video games). The emergence and dominance of the label “gamer” to describe someone (usually young and male; Duggan, 2015) who spends long amounts of time playing digital games points to the widespread dominance of digital games in contemporary culture, as does the presence of (digital) game devices in over 90% of Australian households (Brand et al., 2019). Constant forecasts of the growth of the digital games (again, usually referred to as just “games”) software and hardware industry (Merel, 2017), as well as related industries such as esports (Kelly, 2018), have been used to bolster claims that digital games are the foundation of current Internet technologies and have played a role in preparing the human race for the new age of human–computer interaction (Meeker, 2017). Digital games are promoted, seemingly without consideration of the other types of games that precede and exist alongside them. In a reflection of this trend, many calls for gamification for health use the term “game” while describing only digital games. While recent major industry reports have promoted (digital) games as “the most engaging form of social media” (Meeker, 2017, p. 114), they do not mention that the majority of humankind likely grew up playing games, both alone and with their peers, and that games and play are a fundamental cultural force embedded deeply in society (Caillois, 1958/2001). Games (and gamification by extension) cannot be understood without first examining the characteristics and intricacies of the game form.

The term “game” is notoriously difficult to define (Stenros, 2016). In his review of definitions of this term, Stenros (2016) identifies the common themes they share: rules; purpose; duality of being artifacts and activities; players; productivity; separation from the world; conflict; and telicity (leading to a definite end). Stenros demonstrates that considerable debate and even opposing positions on each of these themes exist, but also that it is this debate that shows how important these themes are when conceptualizing games. From Stenros’ synthesis of his findings, it is clear that games are much more than digital games, or even other forms of predigital games, such as board games, ball games, or word games. As tools of leisure, challenge, and simulation (for example, in the form of gambling, meritocracy, and entertainment, respectively), games and game-like processes are a cultural construct that have served a wide variety of purposes in human society for millennia (Caillois, 1958/2001). When creating gameful experiences, gamification designers should therefore draw inspiration from not just digital game elements, but also these broader sociocultural constructs.

According to French sociologist Roger Caillois (1958/2001), there are four types of play: *agon* (competition), *alea* (randomness and uncertain outcomes), *mimesis* (imitation; or pretending to be, or act for, someone or something else), and *ilinx* (the exhilaration of vertigo, for example, via dancing or riding roller coasters). While not all types of play are present in every game, every game contains one or more of these types of play. Referring to this framework, one can see that most mainstream applications of gamification, such as the “PBL triad” (points, badges, and leaderboards; Chou, 2015)—as well as commonly mentioned game elements such as progress markers, achievement-based rewards, and so on—rely mostly on *agon* (Sicart, 2014; Idone Cassone, 2016). There is much room for designing and implementing gamification that takes advantage of the appeal of *alea*, *mimesis*, and *ilinx*, particularly in a way that supports intrinsic motivation (Helmefalk et al., 2020) and promotes innate satisfaction with the activities the gamified service is intended to encourage (Sicart, 2014).

### Reflections on How Gamified Systems Communicate Through Procedural Rhetoric

Games represent, but are also *separate* from, the world around them: they are a “voluntary safe action” with “slight consequentiality” (Deterding, 2013). This “pretend context” allows for safer rehearsal of emotional regulation (and other types of adaptive regulation) strategies (Granic et al., 2014), and can also serve educational purposes, for example, by allowing exploration of complex situations (Schrier, 2017). However, as games reflect the world around them (Stenros, 2016), like other works of fiction they are inherently biased toward communicating certain views or beliefs, whether directly via plot/narrative, indirectly via premise, setting, and visual representations, or procedurally via available actions such as rules and mechanics (Juul, 2013). The same applies for gamified systems, including gamified health technologies. For example, by only providing functionality to record performance metrics (i.e., distance, duration, and location), and rewarding based on these metrics, the Nike+ system implicitly communicates that other enjoyable aspects of running, such as the runner’s high, or the mindful interaction between human and environment, are less important (Sicart, 2014). This can lead to users feeling pressured to log those types of data, potentially at the expense of what the user may instead personally find meaningful about running, and compromise intrinsic enjoyment of, and motivation to engage in, the activity (Schmidt-Kraepelin et al., 2019). In short, these applications of gamification reward users for appearing to have done the behavior, rather than the behavior (and its intrinsically enjoyable aspects) itself.

Games and gamified systems necessarily depict real-world processes through processes of abstraction, analogy, and imitation (Juul, 2013; Idone Cassone, 2016). These processes can range from simple, abstract loops of achievement and reward (e.g., completing a task to earn points), to more concrete experiences that vary depending on the type of game. For example, a cooking game could depict “preparing spaghetti with meatballs” and ask its players to stir the sauce by drawing circles on the screen. Games and gamified systems relating more directly to mental health and wellbeing could depict a wide variety of experiences to varying levels of abstraction, such as “reframing a thought,” “a day with severe depression,” “injecting heroin intravenously,” or “managing a panic attack.” Players of these games, and users of these gamified systems, can interact with these represented experiences repeatedly and with less consequence. Given appropriate levels of reflection and critical thinking on the part of the players and users of these systems (Tyack and Wyeth, 2017), this ability to rehearse and explore otherwise distressing or unsafe experiences has potential in supporting the learning of adaptive regulation strategies (Granic et al., 2014), increasing and deepening understanding of complex issues (Schrier, 2017), and even changing attitudes (Bogost, 2007).

However, in the case of complex sociological issues such as mental health and wellbeing, and its intersections with other social categories such as (but not limited to) ethnicity, sexuality, and gender, inappropriate abstraction may unintentionally communicate an undesired message that may undermine technology aims or even harm users. For example, when representing the experience of substance addiction, it would be important to strike a balance between depicting enough of the experience to make it meaningful and abstracting it sufficiently to maintain the clarity of the intended message and the smoothness of the user experience. The ideal user experience should not be offensive to either the player or the group whose experience is being represented. The consultation of all relevant stakeholders (including but not limited to mental health researchers, technology users, clinicians, software developers, and, where involved, game and gamification designers) is crucial for the success of gamification for mental health and wellbeing (Fleming et al., 2016).

McCallum (2012) argues that while game designers can design for player experience, they cannot control it, and that each player is different and may interpret and play the game in ways the designer may not have intended or predicted. The same observation has been made for health technologies in general (Greenhalgh and Russell, 2010; Pham et al., 2016). Therefore, situations where abstract depiction could be difficult, misleading, or otherwise impractical to perform and test may not be suitable for gamification. While it is the role of the designer and testers to anticipate unintended outcomes during the design and development process, this may not be feasible, or appropriate, for all projects. Technology designers facing situations like this should be aware of the problems with applying games to these cases and could consider alternate behavior change strategies and techniques.

Some activities, on the other hand, could be particularly complementary with gamification. As games are artifacts and activities that require active participation (or *play*) to progress, they are a natural complement to skill-building activities or those that require active participation (e.g., exposure therapy; Donker et al., 2018), as well as activities involving direct audiovisual or haptic feedback (e.g., an educational software that uses virtual reality and biofeedback monitoring to support mindful meditation; Choo and May, 2014). Designing technologies to contain more of these types of activities, and integrating gameful design concepts within these activities instead of solely applying them peripherally via progress feedback, points, and rewards (the most commonly applied gamification elements in apps and technologies for improving mental health and wellbeing; Cheng et al., 2019), could result in mental health and wellbeing technologies and interventions that are more engaging and well-received.

## Developing and Evaluating Gamified Technologies for Mental Health and Wellbeing

### The Supportive Role of Gamification

Huotari and Hamari’s (2012) definition of “gamification” implies that how gamification can “enhanc[e] a service” should be considered before it is deliberately implemented “to support [a] user’s overall value creation.” However, the lack of theoretically driven health gamification (Lister et al., 2014) suggests that this does not happen. Instead, gamification is seen by some as “strip-min[ing]” games of their “useful” elements (Ferrara, 2013) and superficially applying them to further pre-existing goals. This approach to gamification is reductionist and implies an assumption that individual gamification elements have additive, instead of synergistic, effects on the system or service being gamified. However, much like how digital health technologies operate within a wider psychosocial context (Ritterband et al., 2009; Greenhalgh and Russell, 2010), so do games operate within a system that players find engaging precisely because all components of the game (not just its individual elements) work together with the player, environment, and potentially other sociocultural factors to create a satisfying player experience (Deterding, 2015). Naturally, this would also apply to gamified technologies for mental health.

Sicart (2014) argues that technologies, particularly gamified technologies, should support a person in achieving “the good life.” Similarly, in their definition Huotari and Hamari (2012) emphasize the supportive role of gamification. Considering their definition further leads to the conclusion that to maximize effectiveness, gamified technologies should be intentionally implemented to support their *users* (people), *value* (evidence-based processes), and the *creation* of this value (user interaction with these evidence-based processes). A high-level amalgamation of the Internet Interventions Model with instructional design principles (Hilgart et al., 2012) can be used as a base from which to visualize the development of health technologies. On this model, users, value, and value creation would map roughly onto the *analysis* (user) and *strategy* (mechanisms of change and website use) phases: namely, identifying needs, formulating goals, and developing strategies to achieve those goals.

#### Gamification Supporting Users (People)

The Internet Intervention Model lists seven characteristics users differ on that could influence how they interact with the intervention: disease type and severity; demographics; psychological traits; cognition; attitudes and beliefs; physiological factors; and skills (Ritterband et al., 2009). The importance of tailoring user experience for both user satisfaction (Cheng et al., 2018) and improved clinical outcomes (Tregarthen et al., 2019) has been established. This can range from relatively simple (e.g., the system consistently referring to the user with the right name and pronouns after a user inputs them) to more sophisticated tailoring based on user behavior and preferences. Whether or not these are considered gamification elements, both passive, system-driven tailoring (or “personalization”) and active, user-driven tailoring (or “customization”) are among the most commonly implemented elements in gamified apps and technologies for the improvement of mental health and wellbeing (Cheng et al., 2019). The endorsement of both personalization and customization suggests that not only do users of these technologies want technology to support them, they also want to *help* the technology support them.

Researchers have suggested that gamification designers could consider incorporating cooperative mechanics (Helmefalk et al., 2020), thereby supporting connections between users. This could be achieved by drawing inspiration from contemporary digital games (Cheng et al., 2019). One example is *Journey* (Thatgamecompany, 2012), whose complete focus on cooperative social mechanics contrasts that of many mainstream digital games and typical cases of social gamification. *Journey’s* multiplayer mode encourages social cooperation by making the game easier to play when playing with another player (given a sufficiently cooperative partner). Importantly, while multiplayer mode can make it easier to solve puzzles, it is not necessary to progress in the game. By making social cooperation optional, *Journey* preserves player autonomy and prevents its players from potentially being frustrated by circumstances they cannot control. Furthermore, instead of invoking social status and competition through elements such as badges, levels, leaderboards, and customizable avatars (usually with prestige markers such as special clothing items or accessories), *Journey* removes overt markers of difference such as language and gender, reflecting a more egalitarian philosophy of prioritizing current actions over previous achievements. This may align better with the goals of mental health and wellbeing interventions.

#### Gamification Supporting Value (Evidence-Based Processes)

Many mental health and wellbeing technologies, particularly those with an academic origin, adapt existing evidence-based therapies and techniques. For example, Earle et al. (2018) describe an adaptation of personalized normative feedback for problematic alcohol use, while Vella et al. (2018) describe a gamified wellbeing app that adapts techniques from acceptance and commitment therapy and positive psychology. However, previous research has identified a lack of application of health behavior change theory in gamified health and wellbeing technologies (Lister et al., 2014; Schmidt-Kraepelin et al., 2020). Furthermore, applications of gamification for health and wellbeing also appear to lack adequate reference to motivational theory (Johnson et al., 2016).

Therefore, psychological and health behavior change theories, including but not limited to self-determination theory (Deci and Ryan, 2000) or the behavior change wheel (Michie et al., 2011), should also drive the implementation of gamification for mental health and wellbeing. Designing gamified technologies with a focus on satisfying the innate psychological needs specified by self-determination theory can promote motivation that is relatively more internally regulated, and create conditions favorable for psychological wellbeing (Ryan and Deci, 2000). This could complement self-management interventions, which are a cost-effective, autonomy-promoting method of improving mental health outcomes that are helpful for people with serious mental illness (Lean et al., 2019). The choice of theory to apply during design and development would likely vary across contexts (e.g., acute vs. chronic illness; one-off assessment vs. long-term engagement), and an analysis of this context should be conducted in the early stages of gamification design.

Finally, it is important that gamification does not overshadow or distort user motivations for engaging with a health technology, and instead supports the delivery of an intervention’s “active ingredient” (Vella et al., 2018). While this active ingredient can take many forms, such as an intervention principle (Mohr et al., 2015), an app, a technology, or other process, it is important that it has an evidence base showing empirical support for the techniques or mechanisms through which the technology aims to improve its users’ mental health and wellbeing.

#### Gamification Supporting Value Creation (User Interaction With Evidence-Based Processes)

While it is crucial that a gamified health technology draws on evidence-based theories and techniques, to ideally promote an improvement of health-related outcomes, no promotion will occur if the technology is not used. Therefore, gamified apps and technologies should also support the creation of value, or the direction of user effort toward the abovementioned evidence-based processes.

When considering gamified technologies at face value, there is a tension between being easy to use via good user experience design, and being sufficiently challenging to be motivating via good game design (Deterding, 2015). Furthermore, while immersion and similar flow-inducing techniques have been cited as advantages of gamification (Baranowski et al., 2008; Helmefalk, 2019), the somewhat opposite approach of encouraging mindful, active self-reflection may also be conducive toward achieving mental health goals, for example, through learning and understanding complex situations (Tyack and Wyeth, 2017). Similarly, Cheng et al. (2018) found that participants perceived activities that required more active participation (e.g., creating, and physically typing, a message to a loved one) more helpful and meaningful than activities requiring less participation and effort. While gamified technology designers should focus on making it easy and intuitive for users to navigate certain parts of a technology (e.g., registration and setup), they can also consider where, and how, it may be appropriate to make activities more challenging. As game developers are experts in designing challenges, this seems a natural area about which to consult their expertise. A collaboration between health researchers, game studies academics, and game developers could potentially lead to a novel, engaging, and effective intervention for mental health and wellbeing.

### Designing and Developing Gamified Technologies

So far, this article has outlined themes to consider when creating gamified technologies for mental health and wellbeing. However, how should this be executed in practice?

#### Briefly Reviewing Gamification Design Methods and Frameworks

In their systematic review on gamified apps and technologies for mental health and wellbeing, Cheng et al. (2019) provide a taxonomy of gamification elements that mental health technology developers may find a helpful frame of reference. Similarly, Helmefalk (2019) lists a number of psychological mediators synthesized from gamification articles across seven disciplines, and proposes that “gamificators,” or people who gamify, consider mechanics, psychological mediators, and desired outcomes (M-PM-O) when creating a gamified technology. Additionally, Ašeriškis and Damaševičius (2014) list a number of common “gamification patterns,” or design patterns commonly found in gamified systems, that can be used as reference when designing resource systems (such as for points) within gamified apps. For a more macroscopic view of the development and evaluation process, Floryan et al. (2019) merge general gamification principles with the Internet Intervention Model. Finally, Deterding (2015) describes a method of gameful design that starts with considering the core goals of the activity and brainstorming how the challenges inherent to that activity can either be removed or have motivational affordances created to support it.

A systematic review of gamification design frameworks by Mora et al. (2017) identifies iterative processes, user-centered design principles, and psychological and motivational theories (such as self-determination theory) as key principles shared by the majority of reviewed design frameworks. Mora et al. (2017) also identify common game design elements specified by these frameworks, with the most common being objectives, rules, social interaction, and fun, and the application of these elements to effect a desired behavior. However, as described above in the section “Understanding Gameful Experiences Through Understanding Games,” more recent research has shifted to explicitly consider users of gamified systems as active participants in the creation of a gameful experience (Landers et al., 2019). To that end, some psychometric scales measuring perceived gameful experience have been developed, such as GAMEX (Eppmann et al., 2018) and GAMEFULQUEST (Högberg et al., 2019), and researchers should select which instrument suits their objectives better. As GAMEFULQUEST does not explicitly measure negative affect (or lack thereof), GAMEX may be more appropriate for contexts where this would be relevant (for example, if designers want to design an experience void of negative affect). Regardless of which instrument is ultimately selected, it will be crucial for future gamification research to account for gameful experience and confirm that the gamified technologies under investigation are actually perceived as such.

Previous research has noted that evaluations of gameful experience should only be conducted when the technology reaches a certain maturity (Morschheuser et al., 2017). Early in the design and development process, it may be more helpful to obtain richer data directly from the target end user, for example through qualitative methods. While standard software design processes (e.g., the use of personas, user journeys, and A/B testing; Morschheuser et al., 2017) allow for the consideration of user perspectives, when dealing with sensitive and highly personal topics such as mental health, more equitable methods that allow for the direct contribution of rich data from the end user in an empowered context may also be needed.

#### Iterative Design Through Participatory Design Methods

Participatory design (PD) and other co-design methodologies are gaining traction in eHealth and mHealth, particularly for mental health and wellbeing. Simply put, these methodologies involve target end users in the design, development, and evaluation processes of technologies and interventions. While the concept of user testing is not new, and calls for applying gamification for health and wellbeing also include recommendations to test that this application is appropriate (Cugelman, 2013), a key tenet of PD is that the target end user should be present at *all* stages of the design, development, and evaluation process. This prevents their tokenistic involvement either too early or too late in the process to achieve real impact (Orlowski et al., 2015). Involving end user populations at early stages of development, for example, via evaluation of wireframes, prototypes, and design concepts (Ospina-Pinillos et al., 2018), can also help ensure that resources are not wasted on inappropriate solutions. In Australia, PD has been emphasized as a key strategy for the development of evidence-based interventions, particularly for youth mental health (Hagen et al., 2012).

Co-designing technologies that promote autonomy with healthcare consumers, particularly people with lived experience of mental illness, can also contribute toward counterbalancing their frequent experiences of unidirectional, paternalistic doctor–patient relationships. PD can help designers learn directly from their target end users how best to present and structure technologies for mental health and wellbeing, including content, tone, frequency, and module length, if applicable (Fleming et al., 2016). When brought to its natural extension, this co-design process places target end users at the center of the process, allowing them to directly contribute to, or specify guidelines for, developing the technology. These end user guidelines can then be considered in tandem with evidence-based best practice. PD has been found to be an important and effective way of making sure that technologies are as current and suited to the target population as they can be (Ellis et al., 2014). Furthermore, as PD spans multiple phases (from the start to the end of the project), it can be conducted with a variety of research methodologies, including focus groups, PD (and co-design) workshops, surveys, and user testing (Hagen et al., 2012; Ospina-Pinillos et al., 2018). This triangulation of methods can help support the validity of the ensuing findings.

Participatory design can also be instrumental in reflecting the priorities and concerns of the target end user population into technologies designed with them, particularly those who have historically been marginalized (Hagen et al., 2012)—including but not limited to those with diverse genders and sexualities, First Nations peoples, and culturally and linguistically diverse people—as well as those who otherwise experience a sociological power imbalance such as children (Yarosh and Schueller, 2017). Similarly, as people with chronic conditions (including mental illness) are experts in their own experience, PD can facilitate the contribution of this lived experience to directly influence the development of technologies for people like them in contextualized and rich detail (Jessen et al., 2018). This is particularly important given that multiple forms of marginalization intersect to create compounded barriers to accessing mental health resources (Brown et al., 2016a). PD can also help confirm that the development of a particular technology is appropriate for the target population’s needs, particularly those who face barriers to seeking information or care, such as mental health stigma (Ellis et al., 2014). In cases where resources (including time and funds) are limited, PD may also be an efficient way of both identifying the best solution given adequate communication of these constraints, as well as reflecting the concerns of the target population back to other stakeholders such as health services. Notably, through the use of PD methodologies with veteran counseling service Open Arms (including veterans, health professionals, and administrative staff), LaMonica et al. (2019) were able to identify areas of the service pathway that could be improved, leading to rapid service change.

Participatory design has also been successfully used for applying games to mental health and wellbeing. Through using a PD methodology named “experiential participatory and interactive knowledge elicitation,” Sockolow et al. (2017) were able to obtain feedback on their proposed mHealth game’s storyline from their target audience (13–17-year-old African American young women from under-resourced communities). Specifically, through engaging with these young women, the authors were able to identify aspects of their prototype that their intended audience found off-putting (including background images, character body types, skin tone, and slang) and act on their participants’ suggestions, increasing the credibility of the game with the target audience and the likelihood that they would play it. Though Sockolow et al. report on the development of a serious game and not a gamified technology, a similar process for a gamified technology could elicit insights into unforeseen problems with the technology, brainstorm methods on how to address these problems, and confirm the acceptability of the technology.

Finally, previous research shows the importance of bringing all stakeholders together—those with lived experience of mental illness (service users), those who deliver the care (health professionals and service workers), and those who study the phenomena (mental health researchers)—allowing all stakeholders to have an active, unique contribution to the final end product (Ospina-Pinillos et al., 2018; LaMonica et al., 2019). Involving health professionals in the PD process is particularly crucial as while their endorsement is a large motivating factor in encouraging service users to use gamified mental health interventions, health professionals are time-poor and a subset further hold negative attitudes toward incorporating digital technologies into mental health practice (Hopia and Raitio, 2016; Hickie et al., 2019). Naturally, when incorporating applied games into mental health technologies, those who build and play games (game developers and players) should also be included in PD processes.

### Clinically Evaluating Technologies in Tandem With Software Development Schedules

Digital technologies are not cheap to develop. Furthermore, eHealth/mHealth research teams are often small and work on projects with strict time limits defined by funding bodies. Hence, it is important to maximize the temporal and financial efficiency of research collaboration with software developers when producing technologies for mental health and wellbeing. While it is necessary for developers to accommodate research practices (e.g., the relatively longer length of clinical evaluation trials compared to user research studies), the best outcomes arise when researchers accommodate software development practices as well, such as quick production cycles and the iterative improvement of a Minimum Viable Product (Fleming et al., 2016).

The traditional gold standard of clinical evaluation, the randomized controlled trial (RCT), was originally developed to evaluate drugs. In order to reduce possible confounders, RCTs adopt restrictive inclusion and exclusion criteria and sophisticated blinding procedures to isolate and identify causation effects. However, unlike drugs, health technologies are psychosocial and are necessarily embedded into a wider social context (Greenhalgh and Russell, 2010). By viewing these contextual factors as confounders, RCTs undermine the complex mechanisms through which health technologies operate (Pham et al., 2016). Instead, eHealth and mHealth researchers have recommended using evaluation methods that allow for reflexivity and the consideration of contextual factors such as stakeholder interactions and power dynamics (Greenhalgh and Russell, 2010), as well as rapid methods that accommodate the naturalistic factors of technology usage and the iterative nature of technological development (Mohr et al., 2015). Depending on the nature of available data, these designs can be strengthened by incorporating more rigorous research design elements, such as case-control matching. Instead of viewing external factors as confounders, eHealth and mHealth research and researchers should reflexively acknowledge how these factors could both weaken and strengthen their research conclusions (Greenhalgh and Russell, 2010).

A key strength of the RCT is that causal relationships between the intervention being tested and the clinical outcomes under investigation can be established. However, in traditional RCT designs, this means that the intervention is “locked down” into an unchanging state and that all RCT findings relate to this state. For technological interventions, this often means that the intervention has long become obsolete by the time the RCT concludes and findings are published. To mitigate this problem while still preserving scientific rigor, Mohr et al. (2015) suggest evaluating “intervention principles” instead of the intervention itself, with this approach allowing iterative improvement of the intervention under investigation. Naturally, in order to provide future researchers with adequate knowledge of the context surrounding the intervention, any modification to the intervention must be comprehensively reported (Mohr et al., 2015).

**FIGURE 1:**
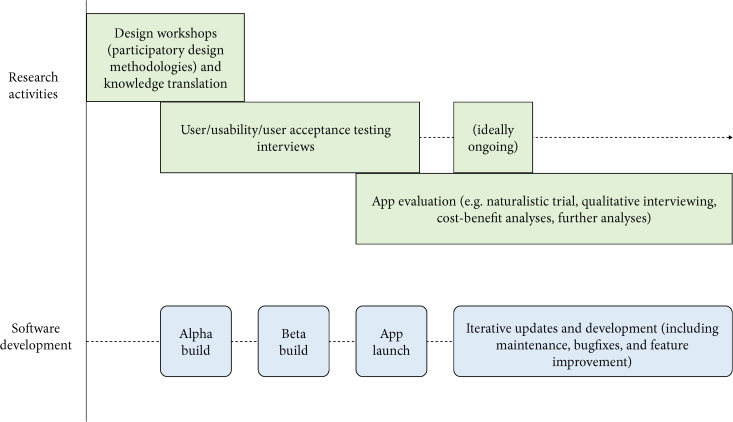
A possible model for developing and evaluating mHealth apps.

Figure 1 shows a complete co-design, development, and evaluation process of a gamified mental health and wellbeing app that was adopted by a doctoral project (Cheng, 2019). Importantly, as researchers conducted user testing across multiple stages of development (two time points before app launch and one time point after app launch), findings fed back into the development process. As valuable insights from representative end users can be obtained during the design and testing process, involving researchers in these activities enables the contribution of this data to the literature, potentially through rigorous qualitative methodologies such as thematic analysis or grounded theory. Furthermore, the use of both qualitative and quantitative evaluation methodologies takes advantage of the strengths of each approach, and enables the triangulation of, and a higher level of confidence in, project findings.

This model identifies and delineates the individual contributions of research and industry. However, while executing this process we encountered multiple complexities. During design workshops and user testing, representative end users expressed preferences that would often be unreconcilable with existing project budget and technological infrastructure. It may have been helpful to have the software developers take part in the PD workshops alongside the representative end users. However, their presence may also have influenced workshop dynamics, as their expertise in technology development would have placed them in a relatively higher position of authority, potentially undermining the workshop’s participatory nature. This dynamic would have to be managed and carefully balanced by workshop facilitators. Importantly, although some preferences were unable to be implemented, they were still captured and disseminated for future reference (Cheng et al., 2018).

## Recommendations for Implementing Gamification for Mental Health and Wellbeing

In the first section, this conceptual analysis briefly reviewed the literature on gamification for mental health and wellbeing. Then, in the next section, “Interrogating Gamification for Mental Health and Wellbeing,” it examined the complexities of the game form to demonstrate that gamification is not just implementing surface-level game mechanics such as points, badges, or leaderboards. In the following section, “Developing and Evaluating Gamified Technologies for Mental Health and Wellbeing,” this article further explored the process of designing, developing, and clinically evaluating gamified technologies for mental health and wellbeing, through the application of theory, methodologies, processes, and frameworks which are standard in their fields of origin, but rarely combined (as identified by, e.g., Schmidt-Kraepelin et al., 2020).

This section summarizes the previous three sections into brief recommendations for implementing gamification for mental health and wellbeing (Box 1). These recommendations are not intended to stand alone, and the rest of the article should be read to understand the context within which they sit. The aim of these recommendations, and this article in general, is to provide clear guidance for researchers and practitioners interested in applying game design concepts to mental health and wellbeing initiatives, with a focus on advancing the field’s collective knowledge via clear language, unified terminology, the application and evaluation of theory, comprehensive and constant documentation, and transparent evaluation of initiative outcomes.

**Recommendations for implementing gamification for mental health and wellbeing.**

1. Assess the suitability of implementing gamification and make sure it complements the technology’s aims and processes

2. Implement gamification intentionally at a deeper, systemic level to support users, evidence-based processes, and user engagement with these processes

3. Assess the acceptability of the gamified technology throughout the design and development process, involving all stakeholders (including but not limited to representative end users, researchers, health professionals, software developers, and game designers)

4. Evaluate the impact of the gamified technology

5. Provide comprehensive and detailed documentation of the (co-)design, development, and evaluation process, using terminology correctly and consistently

First, the suitability of implementing gamification should be assessed. Before mental health technology designers assess how best to apply gamification to their technology, it is best to assess whether gamification should even be applied at all (see the section “Reflections on How Gamified Systems Communicate Through Procedural Rhetoric”). This assessment should concretely operationalize the intended aims of the technology and consider how gamification can be implemented to support these aims (see the section “The Supportive Role of Gamification”). Furthermore, the purpose(s) of gamification should be determined.

Second, gamification should ideally be implemented at a deeper, systemic level of the technology (see the section “The Supportive Role of Gamification”). Designers should not refer solely to digital game elements, but also draw inspiration from more fundamental characteristics of games, such as the four types of play that underlie the game form (Caillois, 1958/2001), or procedural rhetoric (see the section “Reflections on How Gamified Systems Communicate Through Procedural Rhetoric”). Multiple researchers have proposed methods of implementing gameful design that would suit different needs (Deterding, 2015; Mora et al., 2017; Morschheuser et al., 2017; Floryan et al., 2019), as well as elements and psychological mediators to consider when designing (Cheng et al., 2019; Helmefalk, 2019). Gamification should interact with the other components of the technology to create a coherent system (Deterding, 2015) that supports users’ individual differences and preferences via passive and active tailoring as well as (optional) social connection between users via social mechanics. Cooperative social mechanics may align more with the goals of mental health and wellbeing technologies than competitive social mechanics (Cheng et al., 2019). While the choice of theory should be driven by the context surrounding the gamified technology (e.g., its purpose, whether its target mental health domain is acute or chronic, whether it aims to support one-off or sustained engagement, etc.), the gamified technology should also be deliberately designed to support the evidence-based theories and techniques that inform its content. Examples of such theories could include, but are not limited to, health behavior change theories such as the behavior change wheel (Michie et al., 2011; Lister et al., 2014) or theories of motivation such as self-determination theory (Deci and Ryan, 2000; Ryan and Deci, 2000). Finally, gamification should support user interaction with the technology by directing user effort away from components not directly related to the technology’s aims (e.g., registering an account). Instead, user effort should be directed toward the evidence-based components, through activities that require active user participation and that provide engaging, interesting levels of challenge, naturally complementing the ideals of gameful design.

Third, the acceptability of the gamified technology should be assessed throughout the design and development process. Early testing of key concepts prevents wasted resources on unsuitable concepts and improves the acceptability of a technology with its target audience (Sockolow et al., 2017). Ideally, a mixture of survey and interview (including focus group) methodologies should be used. Empirically validated user experience scales such as the System Usability Scale (Lewis and Sauro, 2009) and the User Engagement Scale (O’Brien et al., 2018) can provide quick measures of user experience that can be compared across time, and PD and related co-design methodologies give all stakeholders the opportunity to contribute their unique expertise to the design process (see the section “Iterative Design Through Participatory Design Methods”). For a gamified mental health and wellbeing technology, stakeholders might include, but not be limited to: representative technology users (e.g., with lived experience of mental illness), health professionals, researchers, software developers, game designers, and game players. While it may be useful to provide opportunities for different stakeholder groups to co-design the technology with each other (e.g., a PD workshop), it is also important to be mindful of the possibility of implicit power dynamics influencing the final outcome. It is also important to confirm the acceptability of what the technology and its gamification may be communicating, to prevent its content and functionality from being misinterpreted and misused.

Fourth, the impact of the gamified technology should be evaluated (see the section “Clinically Evaluating Technologies in Tandem With Software Development Schedules”). Ideally, the technology should be evaluated across multiple stages of implementation so that early findings can be iteratively applied toward making improvements (Figure 1). As above, survey and interview methodologies could be used during initial stages of development (pre-alpha, alpha, and beta) to obtain a mixture of snapshot scores that can be compared across time and rich qualitative data that provides more insights into how to further improve the technology. More comprehensive evaluations of the technology’s impact (relating to the technology’s specific purpose, e.g., users’ depression symptoms, self-efficacy, etc.) could then be conducted at later stages. It is also crucial to assess the subjective level of gameful experience perceived by users of the gamified technology, in order to confirm that the technology has been adequately gamified (Landers et al., 2019; also see the sections “Understanding Gameful Experiences Through Understanding Games” and “Briefly Reviewing Gamification Design Methods and Frameworks”). To accommodate the fast pace of technological change and the complex contexts of technology use, a wider variety of faster and more flexible methods, such as qualitative data collection and analysis, naturalistic evaluation trial designs, and analyzing usage analytics should be employed. A mix of methods and data sources also suits different research questions and enables triangulation of findings with increased convergent validity. As there is little research on the long-term effects of gamification, this should also be evaluated, if possible.

Finally, the design (ideally co-design), development, and evaluation process should be documented comprehensively (see the sections “Briefly Reviewing Gamification Design Methods and Frameworks” and “Clinically Evaluating Technologies in Tandem With Software Development Schedules”). The theories applied and principles evaluated should be defined prior to the start of evaluation, for example, with a principle statement (Mohr et al., 2015), which should describe the purpose and functionality of both the technology and how gamification supports this (assessed and operationalized as part of Recommendation 1) in detail. While many gamified technologies integrate multiple gamification elements, making their individual impact difficult to evaluate (Johnson et al., 2016), detailed documentation of these, and other, design features will facilitate a more accurate interpretation of any resulting outcomes (Mohr et al., 2015). Cheng et al. (2019) provide a suggested taxonomy, developed from existing gamification literature and refined following a systematic review of gamified mental health apps and technologies, for this purpose. While gamified technologies should be conceptualized as a system and should not be reduced to their individual elements (Deterding, 2015), listing individual gamification elements and using terminology consistently increases clarity and gives researchers (and designers) a more complete, accurate picture of the technology described. This is particularly relevant for researchers and designers unfamiliar with the study of games and gamification and who are encountering gamification literature for the first time. As games and play are a fundamental cultural force in society, these researchers and designers would likely have a lay familiarity with games in personal and informal contexts, with the resulting differences in conceptualization and terminology contributing to the inconsistent use of terminology reviewed earlier in this article. In this situation, clear, cohesive literature would contribute greatly toward harmonizing different conceptualizations of gamification.

## Further Directions

The recommendations above provide suggestions for implementing gamification for mental health and wellbeing, summarized from a literature review primarily informed by the medical and game studies literature. While synthesized from the literature reviewed above, these recommendations overlap heavily with existing methods for designing gamification published in the field of human–computer interaction (Mora et al., 2017; Morschheuser et al., 2017). In particular, Morschheuser et al. (2017) also emphasize the importance of iterative design that takes a holistic perspective of the gamified system, a thorough context and user analysis, and sustained evaluation of the solution. As stated above, a higher level of collaboration with industry is needed to develop these gamified technologies. This conceptual analysis also argues for the usefulness and relevance of PD methodologies, personalization (also known as tailoring), and de-emphasis of social status and competition when developing gamified technologies for mental health. Social cooperation features, particularly alongside a complete absence of competition, are rarely present in the design of mental health technologies of academic origin, and the use of gamification is often poorly justified and operationalized in such technologies (Cheng et al., 2019). Future academic work in this area should, therefore, focus on addressing these gaps in research.

Furthermore, it is imperative that the developed technologies be evaluated, not just to determine their effectiveness, but also to evaluate whether the recommendations presented in this article are useful and complement standard research and software development practices. In keeping with latest developments in the study of gamification, evaluations of gameful experience (as recommended by Landers et al., 2019) should also be adopted. More evaluation could also point to which aspects or approaches toward gamification may be more compatible with certain types of technological interventions (for example, a certain approach toward gamification may be particularly compatible with a specific mental health domain or behavioral change mechanism). Furthermore, while gamification has been deployed to support care on an individual (usually self-directed) level, it may be productive to explore the possibility of doing so on a systemic and service-directed level as well. The multilevel model of gamefulness proposed by Landers et al. (2019) that explicitly considers individual users alongside the systems (e.g., organizations) they belong to may be useful in such cases.

More research is also needed to determine the best way to study gamified technologies. While conceptualizing the study of individual gamification elements (through a taxonomical approach) may be more straightforward, games are a system (Deterding, 2015). Subscribing too closely to the taxonomical approach has a danger of implying that gamified systems are made up entirely of the sum of their parts. In addition to measuring gameful experience, the best way forward in this regard may be to apply mixed methods and document both the individual gamification elements (or features) contained in the technology, as well as the broader effect or impact of the technology (potentially through thematically analyzing interviews and focus groups, or even using grounded theory approaches). Naturalistic trial designs are also suitable as they accommodate contemporary software development schedules without necessarily sacrificing research rigor.

## Conclusion

This article synthesizes a conceptual analysis, as well as insights from a complete co-design, development, and evaluation process using a variety of qualitative and quantitative research methods, into recommendations for implementing gamification for mental health and wellbeing. While collaboration with industry is vital for developing gamified technologies, researchers are uniquely positioned to evaluate technologies throughout the development cycle to ensure that the implementation of gamification itself is both acceptable and effective. As gamified mental health technologies represent the intersection of mental health research, human–computer interaction, and game studies, interdisciplinary collaboration, with human–computer interaction and game studies researchers, will be important in answering these questions.

In order for gamification to reflect the cultural artifact it draws its principles from, it is important that future implementations harness more fundamental, but under-utilized, types of play and game mechanics. Games scholars have described games as “unproductive” (Caillois, 1958/2001) and yet “effort[ful]” (Juul, 2005). While there has been much discussion of the high levels of engagement games (and digital games) enjoy despite their unproductivity, perhaps the discourse can be swung to focus, instead, on how to direct the effort games inspire from their players to align with the aims of mental health and wellbeing research.

## Author Contributions

VC conceptualized and wrote the manuscript.

## Conflict of Interest

The author declares that the research was conducted in the absence of any commercial or financial relationships that could be construed as a potential conflict of interest.
